# Connecting education and care: using interdisciplinary college courses to enhance the wellbeing of captive animals

**DOI:** 10.3389/fvets.2026.1853396

**Published:** 2026-07-07

**Authors:** Maryline Bossus, Alison Koons, Brittany Florkiewicz

**Affiliations:** 1 Department of Biology, Lyon College, Batesville, AR, United States; 2 Department of Psychology, Lyon College, Batesville, AR, United States; 3 Research Center for Human-Animal Interaction, College of Veterinary Medicine, University of Missouri, Columbia, MO, United States

**Keywords:** animal enrichment, animal welfare, behavioral husbandry, biology, interdisciplinary education, psychology, student learning, teaching

## Abstract

Interdisciplinary education is a central goal of liberal arts institutions, yet opportunities to integrate different fields, such as biology and psychology, in applied coursework remain limited. Behavioral husbandry, particularly animal enrichment, provides a compelling framework for bridging these disciplines by addressing both the physical and psychological needs of captive animals. Yet, undergraduate offerings of standalone animal enrichment courses are rare in the United States, with most programs embedding enrichment within broader coursework. In response to this gap, a co-taught animal enrichment course was developed at Lyon College, combining biological and psychological perspectives through experiential, project-based learning. Students collaborate with accredited animal care facilities to design, construct, and evaluate enrichment for diverse species, gaining practical skills while contributing to animal welfare. Based on this experience and existing literature, 10 key recommendations are presented to guide course development, including the importance of advance planning, strong partnerships, flexibility, and safety considerations. The perspective also outlines future research directions, such as evaluating impacts on student learning outcomes, animal welfare, and caregiver workload. Animal enrichment courses offer a scalable and impactful model of interdisciplinary education, enhancing student engagement while addressing real-world challenges in captive animal care.

## Introduction

Liberal arts institutions emphasize interdisciplinary education, which involves integrating knowledge, techniques, and perspectives from two or more disciplines (1). This approach enhances students’ ability to understand issues, address problems, and develop innovative solutions beyond the confines of a single field of study (1–3). Interdisciplinary education can involve a variety of avenues, such as the development of majors, minors, and coursework (1). Opportunities for interdisciplinary education between STEM fields, such as psychology and biology, are increasing, with approximately 37% of liberal arts institutions now offering majors in neuroscience and psychobiology (1). One recent study found that an interdisciplinary biology and psychology program (in health psychology) increased student satisfaction, leading to greater success in job acquisition and graduate-level program acceptances (4). While small liberal arts colleges are beginning to offer interdisciplinary majors and minors that bridge biology and psychology, opportunities for interdisciplinary coursework remain limited (1). Such coursework which integrates concepts, and sometimes faculty, from both fields (3, 5) have been shown to be beneficial. For example, an interdisciplinary neuroscience course taught by faculty from both fields increased students’ use of integrative terminology in written reflections (5). However, existing interdisciplinary courses are largely confined to neuroscience and health-related fields, leaving a gap in understanding how this integration functions across other domains. To address this gap, it is essential to develop and evaluate courses that integrate biological and psychological content across a broader range of disciplines.

One suitable field of study for interdisciplinary coursework between biology and psychology is *behavioral husbandry*. Behavioral husbandry requires integrating both physical and psychological wellbeing, with a substantial proportion of behavioral issues linked to past pain experiences (6). In recent years, behavioral husbandry has involved two major types of practices: (1) using training for voluntary participation in veterinary procedures; and (2) developing and implementing enrichment (7). *Enrichment* is a well-documented tool used by caregivers to enhance the biological and psychological wellbeing of their animals (8, 9). Through the use of enrichment, caregivers can reduce negative behaviors, improve physical health (10), all while promoting species-appropriate behaviors (7). In a recent global survey of zoos and their keepers, over 90% of respondents reported that zookeepers are primarily responsible for creating and implementing enrichment activities (11). More complex species often require more diverse enrichment, increasing costs and time researching appropriate enrichment (12). Research is needed to ensure enrichment meets animals’ cognitive, emotional, and social needs while maintaining safety and improving physical health (13, 14). Not all animals have centralized information hubs with best practices for enrichment, making these obstacles more challenging for certain species. Therefore, the two most commonly cited reasons for the limited availability of enrichment in zoos are time constraints and workload for the zookeepers, as well as insufficient funding from the institutions (11, 12). Enrichment is crucial for the welfare of captive animals, but ethical, financial, workload and practical barriers limit its implementation (8, 11, 12).

## Connecting education and animal care

To address the learning needs of higher education students and the limitations faced by captive animal caregivers for essential behavioral husbandry practices, *animal enrichment courses* provide a practical solution. In this perspective piece, the authors share insights gained from their own course, along with findings previously published regarding the University of Tennessee at Chattanooga’s course. The goals are to: (1) provide a list of recommendations for future animal enrichment classes; and (2) suggest directions for future studies on the effectiveness of these programs. In this perspective, it is argued that animal enrichment courses represent an underutilized yet highly effective model of interdisciplinary education that can simultaneously enhance student learning outcomes and address critical gaps in behavioral husbandry practices.

To contextualize the availability of animal enrichment coursework in U.S. higher education, we conducted a targeted audit of official undergraduate course catalogs and course-search pages. We first used College Navigator to generate a master list of associate’s- and bachelor’s- degree-granting 2- and 4-year institutions, with no state restriction. Selected programs/majors included “Agricultural/Animal/Plant/Veterinary Science and Related Fields, Other,” “Animal Sciences, General,” “Animal Sciences, Other,” “Agriculture/Veterinary Preparatory Programs, Other,” “Pre-Veterinary Studies,” “Biology/Biological Sciences, General,” “Animal Behavior and Ethology,” “Wildlife Biology,” “Zoology/Animal Biology,” and “Zoology/Animal Biology, Other.” This search produced 1,698 institutions. Using this master list, we then used ChatGPT Pro to assist with a row-by-row review of official undergraduate catalogs and course-search pages in batches of about 50 institutions. The audit prompt instructed to search for courses related to animal enrichment and to classify each institution as Confirmed Yes, Likely Yes, No Evidence Found, Catalog Inaccessible, Catalog Not Found, or Needs Manual Review. Before assigning No Evidence Found, the prompt required identification of an official catalog or course-search page and searches for the terms “enrichment,” “animal enrichment,” “environmental enrichment,” “behavioral enrichment,” “zoo,” “zookeeper,” “animal training,” “husbandry,” “captive,” “wildlife,” and “animal behavior.” All institutions not classified as No Evidence Found were manually checked against publicly available course information. This approach was necessary because enrichment is often paired with animal training, husbandry, welfare, zoo biology, or captive animal management in course titles and descriptions.

Our examination identified 38 undergraduate courses or course sequences with enrichment-related content, although the level of document student engagement varied. Of these, only 17 clearly indicated as offering clear hands-on activities including designing, building and implementing enrichments, while others appeared to address enrichment primarily through lecture-based instruction, broader animal-care coursework, or zoo-based experiential learning in which the student role in enrichment design or implementation was unclear. Table 1 presents selected examples along this continuum, emphasizing courses with clear applied engagement and a few cases with lower levels of documented engagement. In most cases, enrichment is typically embedded within larger courses or experiential learning opportunities rather than offered as a standalone class. As an example of such a course, in 2015, faculty members from the biology, psychology, and environmental science programs at the University of Tennessee at Chattanooga collaborated with the Chattanooga Zoo, the Tennessee Aquarium, and the Reflection Riding Nature Center to offer an animal enrichment course (15). This course enhanced students’ understanding of the challenges associated with captive animal care and promoted the development of innovative enrichment solutions.

**Table 1:** Selected undergraduate coursework illustrating levels of student engagement with animal enrichment design and implementation in the United States.

Level of documented engagement	Institution	Course	Public course description information
Applied enrichment: design, construction/building, and implementation documented	Lyon College	BIO 309, *Animal Enrichment*	Indicates lecture, enrichment design, building, and implementation.
Applied enrichment: design, construction/building, and implementation documented	Texas Christian University	ENSC/ARST 40553, *Zoo Animal Enrichment*	Indicates lecture, enrichment design, building, and implementation.
Applied enrichment: design, construction/building, and implementation documented	Friends University	ZOSC 320, *Zoo Animal Training and Enrichment*	Indicates lecture, enrichment design, building, and implementation.
Applied enrichment: planning, implementation, documentation, and evaluation documented; construction not explicit	Louisiana State University–Alexandria	BIOL 3541, *Zoo Animal Training*	Describes behavioral husbandry through animal training and environmental enrichment, including goal setting, planning steps, implementing ideas, documenting processes, evaluating outcomes, and revising the process.
Design-oriented enrichment course; building and implementation not documented	Delaware Valley University	SA 2218, *Animal Training and Enrichment*	Includes lecture and enrichment/training design, but does not document student construction or implementation of enrichment.
Zoo-based experiential course with enrichment content; student enrichment design/implementation unclear	Drake University	ENSS 128/BIO 128L /BIO 063L, *Zoo Design and Operations/Zoo Biology Lab*	Mentions captive management, behavioral research, environmental enrichment methods, and experiential learning at Zoo. Does not document applied student design, building, or implementation.
Zoo-based laboratory sequence; student enrichment design/implementation unclear	Santa Fe College	PAZ 1331–2334, *Animal Management Laboratory 1–4*	Zoo-based animal management laboratory sequence, but does not document applied student design, building, or implementation.
Lecture-based enrichment/welfare content	University of California–Davis	ANS 103, *Animal Welfare*	Includes animal welfare/enrichment-related content, but does not document applied student design, building, or implementation.

It is important to note that we referred here specifically to formal college-level courses and did not include enrichment workshops, camps, student organizations, or other informal training opportunities.

The authors of this perspective piece have established an animal enrichment class at Lyon College, a small liberal arts institution in Batesville, Arkansas. This course is offered each spring and co-taught by a biology and a psychology faculty who collaborate to develop course materials that integrate perspectives on animal biology, behavior, and welfare. The authors have collaborations with the Little Rock Zoo (LRZ) and Turpentine Creek Wildlife Refuge (TCWR). The students in this course created and implemented enrichments for 17 different species overall at both facilities since 2024. Enrichment items were requested and approved by the LRZ’s staff while students who worked with the TCWR animals collaborated with caregivers and faculty to develop enrichments. As of 2026, there are 229 institutions accredited by the Association of Zoos & Aquariums and dozens of sanctuaries accredited by the Global Federation of Animal Sanctuaries in the United States, many of which are located near colleges and universities that can also begin to establish similar partnerships and courses. While the benefits of establishing an animal enrichment course can be numerous, there are also some important considerations and drawbacks to keep in mind.

## Recommendations

Based on experiences, 10 key recommendations for establishing a new animal enrichment course or a similar program from an instructor perspective are proposed:

### Plan in advance

Planning ahead for at least one semester, ideally a year, is crucial for creating a collaborative enrichment program (16), which includes identifying potential collaborators, selecting animals, setting goals, coordinating visits, and building and evaluating enrichment items (17, 18). Instructors are encouraged to identify and collaborate with animal care organizations that are accredited by either the Association of Zoos & Aquariums or the Global Federation of Animal Sanctuaries. These organizations set the highest standard for animal welfare and often engage in collaborative educational programming.

### Establish partnerships and expectations

During the development’ stage, it is essential to identify the individuals responsible for evaluating enrichment and clarify whether enrichment ideas will be provided by caregivers or if students will generate their own ideas with close supervision. Formal clarification of goals, roles, and responsibilities can be completed using a Memorandum of Understanding (19) and allow all partners to benefit.

### Consider collaborative course development

Despite the numerous time constraints and busy schedules faced by animal caregivers, it is essential to integrate their insights and perspectives into the structure and content of animal enrichment courses. Caregivers possess valuable knowledge about the specific needs of their animals (20), as well as awareness of the latest trends within their institutions and affiliated accrediting agencies. It also necessitates ongoing communication with animal care staff to ensure that proposed enrichment items are safe, appropriate, and feasible within the facility’s guidelines. In many cases, particularly in accredited zoos, enrichment proposals must meet strict institutional and regulatory standards, which can limit design flexibility and require multiple rounds of review and approval. These constraints can present challenges within the timeline of a single academic semester but also provide valuable insight into real-world processes.

### Encourage and embrace flexibility

A major logistical challenge involves coordinating schedules among faculty, students, and animal care staff. Significant planning is required to organize multiple visits, including initial observations of animal behavior, implementation of enrichment items, and follow-up evaluations. These visits often require the presence of multiple caregivers and may be constrained by institutional priorities, such as peak visitation periods or educational programming at animal facilities. For example, certain times of the year, such as periods with increased school group visits, may limit staff availability and reduce opportunities for student engagement. Anticipating these constraints and planning accordingly is essential for successful course implementation. To address challenges with all parties’ schedules and animals’ availability, it is advisable to have alternative dates for field trips and a variety of prospective animals to work with. Using group work to develop enrichment opportunities can enhance flexibility for students, enabling them to assume roles in which they feel most comfortable.

### Consider institutional constraints

Colleges, universities, and animal care institutions face limitations and challenges, particularly regarding time and funding (11, 12). We recommend engaging in multiple open discussions about financial responsibilities and the availability of caregivers, veterinarians, coordinators, directors, and other personnel involved before officially holding the class. For example, if staff indicate that their availability is limited, instructors may want to reduce the number of field trips or enrichment projects to be implemented. Similarly, if staff express financial constraints, instructors should consider implementing course fees to cover costs for construction materials and field trips. However, this approach could impose financial burdens on students, so it may be necessary to seek low-cost construction materials, grant funding, and donations.

### Foster creativity and resourcefulness

Based on our experience, allowing students to design their own enrichment items, rather than assigning pre-defined designs, provides greater educational benefits. This approach fosters critical thinking and requires students to consider species-specific and individual-specific constraints, including behavioral needs, safety requirements, and enclosure limitations. However, if students are allowed to be creative and develop their own items, these items should be thoroughly checked for the safety and wellbeing of the animals by both instructors and animal care staff.

### Promote additional partnerships

**Figure 1:**
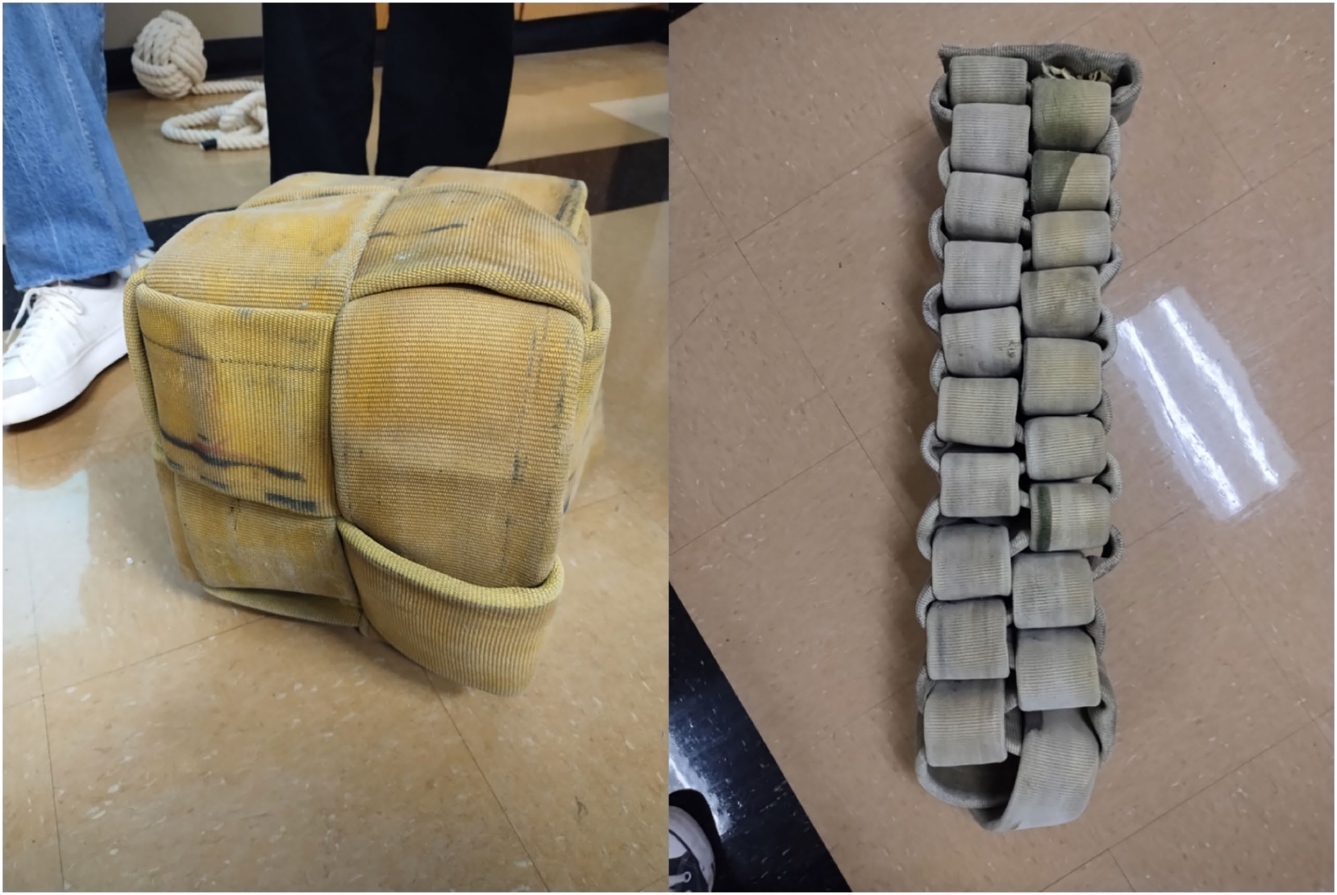
Photos of enrichment items created by students in spring 2025 for zebras and ostriches at the Little Rock Zoo (LRZ). Both items were made from fire hoses donated to the LRZ by a nearby fire station. Nuts and bolts were purchased in bulk from a hardware store, helping to reduce material costs for the course.

Creating animal enrichment courses increases visibility for the animal organization and builds partnerships with local groups to address material, construction, and financial challenges. For example, partnerships with local manufacturing companies can allow for the construction of more durable enrichment items (e.g., steel structures) when the students do not have the means to build such items at their institution. To obtain large amounts of free used fire hose for larger projects, it is recommended to contact local fire departments, as they are required to replace fire hose after a certain number of years of use (Figure 1).

### Cultivate critical observation

Ensuring the safety of both students and captive animals requires vigilance during construction and implementation. A substantial amount of planning must be dedicated to ensuring the safety of enrichment. All designs should be carefully evaluated to eliminate potential hazards, including sharp edges, choking risks, and materials that could cause injury or impaction if ingested along with undesirable behaviors. Close supervision will minimize injury risks and promote the evaluation of these items to guarantee safety and enrichment benefits for the animals. Regularly consult with veterinarians and animal caregivers, providing them with updated information about the enrichment items as students build them, even if the enrichment project was pre-designed and pre-approved (13).

### Celebrate successes and learn from failures

Inform students as early as possible that some animal species may respond negatively to new enrichment items (21). Animals may require time to acclimate before these items can be effectively integrated into their environment, may habituate to enrichment items relatively quickly, or destroy them shortly after they are introduced (14). Emphasizing that troubleshooting is a realistic part of animal caregiving is crucial, and focusing on the progress made in the class can help mitigate disappointment.

### Recruit student experts

Recruiting students who previously completed the course to serve as peer mentors in the future can significantly enhance learning experiences. These student experts can share their prior experiences, provide insights into species-specific needs, and assist with both the design and construction of enrichments. This support is particularly valuable in this type of course where multiple student groups are working simultaneously, potentially at different locations, making it challenging for instructors only.

Many of these recommendations can be directly integrated into course design, including syllabi and associated assignments. Requiring students to generate blueprints or scale models provides opportunities for students to take creative risks while receiving critical feedback from faculty members, veterinarians, and animal caregivers. Finally, implementing a portfolio system where students can document their assignments and progress, along with reflective practice, can foster a deeper appreciation for their efforts and a better understanding of the challenging responsibilities faced by animal caregivers in creating enrichment. Together, these considerations highlight that successful animal enrichment courses require careful integration of pedagogical design, logistical planning, and animal welfare expertise.

## Assessment

There are numerous promising avenues for future empirical research regarding the effectiveness of an animal enrichment course on student outcomes. Future studies should incorporate both quantitative and qualitative approaches, including pre- and post-course assessments, including conceptual understanding, problem-solving skills, and career-related impacts. It is also important to examine how students’ academic backgrounds and career goals influence their engagement and learning outcomes. While an animal enrichment course is likely to attract the most interest from pre-veterinary students and those pursuing other careers with animals, it is crucial to ensure that other students can also benefit from the class, especially if it will be open to a wider variety of students.

There are also many promising research opportunities regarding student-created enrichment for captive animals. It is essential to conduct studies that evaluate the overall impact of student-designed enrichment on animal behavior using empirical methods (22). Such studies should incorporate standardized behavioral metrics, such as changes in activity levels, behavioral diversity, or reductions in stereotypic behaviors. Evaluating the impact of student-designed enrichment across different taxonomic groups can clarify which animals might benefit the most from animal enrichment courses and collaborations. Furthermore, conducting research to compare the effectiveness of constructed enrichment items based on the level of student experience, as well as gathering insights on the most effective enrichment strategies for specific species from various experts (20), would help confirm that animal enrichment courses are valuable for all stakeholders.

Future research should examine the impact of these courses on participating animal care institutions, including potential reductions in caregiver workload, increased enrichment diversity, and improvements in animal welfare outcomes. It would also be valuable to assess the benefits for higher education institutions, including contributions to student recruitment, engagement, and retention, particularly among students interested in animal-related careers.

## Conclusion

The field of behavioral husbandry, including the development, implementation, and evaluation of enrichment presents a significant opportunity for liberal arts institutions to develop multidisciplinary coursework that integrates the fields of biology and psychology. The establishment of animal enrichment courses can enhance student learning while addressing real-world challenges in captive animal care, as well as alleviating the financial and time constraints faced by animal caregivers. Although there are several considerations for designing an animal enrichment course and establishing collaborative partnerships with institutions accredited by the Association of Zoos & Aquariums or the Global Federation of Animal Sanctuaries, the benefits are likely to outweigh the potential challenges. Animal enrichment courses represent an underutilized yet scalable model that can be adopted across a wide range of institutions with access to animal care facilities.

## Data Availability

The original contributions presented in the study are included in the article/supplementary material, further inquiries can be directed to the corresponding author/s.
